# Identification of Important Proteins and Pathways Affecting Feed Efficiency in DLY Pigs by iTRAQ-Based Proteomic Analysis

**DOI:** 10.3390/ani10020189

**Published:** 2020-01-22

**Authors:** Jie Wu, Xingwang Wang, Rongrong Ding, Jianping Quan, Yong Ye, Ting Gu, Zheng Xu, Enqin Zheng, Gengyuan Cai, Zhenfang Wu, Ming Yang, Jie Yang

**Affiliations:** 1 National Engineering Research Center for Breeding Swine Industry, College of Animal Science, South China Agricultural University, Guangzhou 510642, China; wujiezi163@163.com (J.W.); wangxw@stu.scau.edu.cn (X.W.); dingrr@stu.scau.edu.cn (R.D.); qjp_scau@outlook.com (J.Q.); yinhun0517@163.com (Y.Y.); tinggu@scau.edu.cn (T.G.); stonezen@scau.edu.cn (Z.X.); eqzheng@scau.edu.cn (E.Z.); cgy0415@163.com (G.C.); wzfemail@163.com (Z.W.); 2 College of Animal Sciences and Technology, Zhongkai University of Agriculture and Engineering, Guangzhou 510225, China

**Keywords:** feed efficiency, iTRAQ, small intestine, DLY pig

## Abstract

**Simple Summary:**

Feed efficiency is one of the most valuable economic traits in the pig industry. The small intestine is the site where most of the nutrients are absorbed from ingested food. Here, we studied the relationship between small intestinal proteomics and feed efficiency in Duroc × (Landrace × Yorkshire) pigs, which is the most popular commercial pig in the Chinese pork market. Exploring the molecular mechanisms of feed efficiency will create great value for the pig industry. Our research provided a reference for further understanding of the key proteins that affect small intestinal microvilli formation and the important pathways related to feed efficiency in pigs.

**Abstract:**

Feed efficiency is an economically important trait controlled by multiple genes in pigs. The small intestine is the main organ of digestion and nutrient absorption. To explore the biological processes by which small intestine proteomics affects feed efficiency (FE), we investigated the small intestinal tissue proteomes of high-FE and low-FE pigs by the isobaric tag for relative and absolute quantification (iTRAQ) method. In this study, a total of 225 Duroc × (Landrace × Yorkshire) (DLY) commercial pigs were ranked according to feed efficiency, which ranged from 30 kg to 100 kg, and six pigs with extreme phenotypes were selected, three in each of the high and low groups. A total of 1219 differentially expressed proteins (DEPs) were identified between the high-FE and low-FE groups (fold change ≥1.2 or ≤0.84; *p* ≤ 0.05), of which 785 were upregulated, and 484 were downregulated. Enrichment analysis indicated that the DEPs were mainly enriched in actin filament formation, microvilli formation, and small intestinal movement pathways. Protein functional analysis and protein interaction networks indicated that RHOA, HCLS1, EZR, CDC42, and RAC1 were important proteins that regulate FE in pigs. This study provided new insights into the important pathways and proteins involved in feed efficiency in pigs.

## 1. Introduction

Feed cost accounts for 60–70% of total pig production costs [1]. Feed efficiency (FE) is one of the most important economic traits of pig breeding. Currently, feed conversion ratio (FCR) and residual feed intake (RFI) traits are often used to measure FE traits [2]. The heritabilities of RFI and FCR traits range from 0.14–0.40 and 0.30–0.47, respectively [3,4,5]. As a complex economic trait, FE is affected by genetics and environmental factors [6]. Low FCR and RFI ratios correspond to high FE [7]. Previous studies have found that low-RFI pigs have more effective and coordinated immune capabilities than high-RFI pigs. Part of the explanation for this phenomenon may be due to the stronger intestinal endotoxin detoxification ability, increased intestinal *lactobacillus*, and higher antibacterial enzyme activity of low-RFI pigs [8,9]. Besides, Duroc × (Landrace × Yorkshire) (DLY) hybrid pigs have the largest sales share in the Chinese pork market due to their fast growth and good meat quality [10]. Thus, improving the feed efficiency traits of pigs is a significant strategy to achieve an economic gain in the swine industry.

Previous studies have explored mechanisms affecting FE from different levels, including genome, transcription, and protein levels. Many studies have used genome-wide association studies (GWAS) to discover single-nucleotide polymorphisms (SNPs) and candidate genes affecting FE traits [11,12,13]. However, it is not comprehensive enough to study FE only at the genome level. Transcriptomics can analyze FE traits from the pathway level. Transcriptomic studies have found many pathways related to FE traits in pigs and have revealed the molecular mechanisms of FE, including cyclic adenosine monophosphate (cAMP) metabolic processes, oxidative stress, lipid metabolism, and hormone regulation of digestion and absorption [14,15,16]. Even with a large amount of research on FE traits, it should be noted that the main biological process information is mainly explored from the protein level [17]. Moreover, complex posttranslational modifications of proteins, subcellular localization or migration of proteins, and protein-protein interactions are directly related to protein function [18,19]. Therefore, studying feed efficiency traits at the protein level can more clearly explain the mechanism of change of a given trait under physiological or pathological conditions [20,21]. Isobaric tag for relative and absolute quantification (iTRAQ) is a relatively new proteomic quantitative technology that enables simultaneous labeling and relative quantification of multiple samples [22]. Because of its high sensitivity and wide application, iTRAQ has become one of the main tools for qualitative and quantitative protein research. Moreover, small intestine tissue is the most important organ for digestion and nutrient absorption. However, there are few studies on the association between proteomics and FE traits in the small intestinal tissues of pigs, especially in DLY pigs. In the present study, we used iTRAQ-based proteomic analysis to identify differentially expressed proteins in the small intestines of high-FE and low-FE DLY pigs. The aim of this study was to broaden our current knowledge of the molecular mechanisms of feed efficiency in pigs.

## 2. Materials and Methods 

### 2.1. Animals and Samples

The 225 female DLY pigs in this study were all provided by Guangdong Wen’s Foodstuffs Group Co., Ltd. (Yunfu, China). The ethics committee of South China Agriculture University (SCAU) (Guangzhou, China) approved this study (Approval number SCAU#0017). Various phenotypic data for the test pigs, such as the time, feed intake, and body weight of each meal, were measured and recorded by an Osborne Feed Intake Recording Equipment (FIRE) Pig Performance Testing System (Osborne, KS, USA). Throughout the experiment, the test pigs were given free access to food and water in an environmental monitoring room. A total of approximately 12 w was spent recording phenotypic data for each individual’s body weight (BW) from 30 kg to 100 kg. The detailed information of experimental animals is listed in Supplementary Table S1. The FCR was calculated per individual during the trial. RFI was calculated using a method similar to Cai et al. [23]. In the model, predicted daily feed intake (DFI) was estimated using linear regression of DFI on mid-test metabolic BW (MBW), average daily gain (ADG) from 30 to 100 kg, and back fat (BF).

MBW = [(BW at on-test + BW at off-test)/2]^0.75^

At the end of the trial period, we obtained 24 sampling candidate groups of the highest and lowest FE groups from the sorted 225 pigs; each group selected 12 pigs for slaughter. Finally, three pigs were randomly selected from each group for iTRAQ analysis. Then, pigs were euthanized via a captive bolt, followed by exsanguination. Immediately after euthanasia, segments of intestine tissue, which were 50 cm away from the cecum, were collected and flushed with saline. Collected tissue was snap-frozen in liquid nitrogen and stored at −80 °C. The six groups of samples were labeled as HE1, HE2, HE3, LE1, LE2, and LE3. All methods and operations in this study were conducted in accordance with the guidelines of the Animal Protection and Use Committee of South China Agricultural University. All trial protocols were approved by the committee.

### 2.2. Protein Preparation and iTRAQ Labeling

For iTRAQ analysis, the small intestine samples were ground into small pieces and placed in a 1.5 mL centrifuge tube. A 5-mm magnetic bead and an appropriate amount of lysis buffer 3 (1 mM phenylmethanesulfonyl fluoride (PMSF), 2 mM EDTA, and 10 mM dithiothreitol (DTT)) were added. Subsequently, the tube was shaken with a tissue grinder for 2 min (power = 50 HZ, time = 120 s). The protein was separated by centrifugation at 25,000 g for 20 min at 4 °C, and the supernatant was retained. DTT (10 mM) was added to the supernatant and heated in a water bath at 56 °C for 1 h. After returning to room temperature, 55 mM iodoacetamide (IAM) was added, and the tube was incubated in the dark for 45 min. Four volumes of cold acetone were then added, and following incubation at −20 °C for 2 h, the step of adding cold acetone was repeated until the supernatant was colorless. The supernatant was discarded under the same centrifugation conditions. The above steps were repeated: magnetic beads and lysis buffer 3 were added, and the tube was shaken and centrifuged. The supernatant was then taken for subsequent quantification. Next, 100 μg of protein solution from each sample was taken and treated with trypsin enzyme at 37 °C for 12 h (protein: trypsin ratio of 40:1). After digestion, the sample was dried in a centrifugal vacuum concentrator. Next, the samples were redissolved in 0.5 M tetraethyl-ammonium bromide (TEAB) and added to the iTRAQ labeling reagent. The three high-FE samples were 113, 114, and 115 iTRAQ tags, respectively, and the low-FE samples were 116, 119, and 121 iTRAQ tags, respectively.

### 2.3. High pH Reverse Phase Fractionation

The sample was passed through an LC-20AB liquid phase system (Shimadzu, Kyoto, Japan); the separation column was a 5 µm 4.6 × 250 mm Gemini C18 column for liquid phase separation. The dried peptide sample was reconstituted with 2 mL of buffer A (5% acetonitrile (ACN) pH 9.8). After the injection, buffer B (95% cyanomethane (CAN), pH 9.8) was used to elute at a flow rate of 1 mL/min. According to the characteristics of the protein, combined with the chromatographic elution peak map, the samples were combined to obtain 20 fractions.

### 2.4. Liquid Chromatography (LC)-Tandem Mass Spectrometry (MS/MS) Analysis

The combined samples were reconstituted with buffer A (2% CAN, 0.1% formic acid (FA)) and centrifuged to obtain a supernatant. The supernatant was separated by LC-20AD (Shimadzu, Kyoto, Japan) nanoliter liquid chromatography. Buffer B (98% ACN, 0.1% FA) was then used to separate at a flow rate of 300 nL/min for 65 min. The peptides separated by the liquid phase were ionized by a nanoESI and then passed to a Q-Exactive (Thermo Fisher Scientific, San Jose, CA, USA) system for data-dependent acquisition (DDA) mode detection. The MS1 spectra were collected in the range of 350–1600 *m*/*z* with a resolution of 70,000. The MS2 spectra scan initial fixed value was a fixed value of 100 *m*/*z* with a resolution of 17,500. The top 20 precursors with a charge state of +2–+7 and a peak intensity of over 10,000 were selected, and then the sample was subjected to the second round of mass analysis.

### 2.5. Data Analysis

The raw MS/MS data were converted to MGF format by Proteome Discoverer 1.4 (Thermo Scientific, Waltham, MA, USA). Then, the transferred MGF files were identified by the software Mascot (version 2.3.02) and aligned with the protein sequence database to identify the protein. The search parameters for Mascot were as follows: type of search: MS/MS ion search; fragment mass tolerance: 0.05 Da; peptide mass tolerance: 20 ppm; variable modifications: oxidation (M), iTRAQ8plex (Y); mass values: monoisotopic; fixed modifications: carbamidomethyl (C), iTRAQ8plex (N-term), iTRAQ8plex (K). Thereafter, the results of the search were rescored using iQuant software to increase the authentication rate [24], “HE1/LE1, HE1/LE2, HE1/LE3, HE2/LE1, HE2/LE2, HE2/LE3, HE3/LE1, HE3/LE2, HE3/LE3” were set as the comparison group. The screening criteria for differentially expressed proteins were: fold change ≥1.2 or ≤0.84, *p* < 0.05 [25]. For multiple repeated experimental data, the final differential protein needed to be screened with fold change >1.2 (average of the ratios of all comparison groups) and *p* < 0.05 (unpaired t-test of all comparison groups). After converting the pig protein IDs into gene IDs, gene ontology (GO) annotation analysis and Reactome pathway enrichment analysis were performed using the ClusterProfiler package and the ReactomePA package, respectively [26,27]. Another pathway analysis was performed using the web-based Kyoto Encyclopedia of Genes and Genomes (KEGG, http://www.genome.p/kegg). The rich factor represents the ratio of differentially expressed proteins (DEPs) to all proteins in one pathway. Rich factor = number of DEPs in a pathway or term/number of all proteins in that pathway or term. Additionally, the STRING database and Cytoscape software were used to obtain and visualize protein-protein interaction (PPI) information [28].

### 2.6. RT-qPCR 

To assess the reproducibility of proteomic data obtained by ITRAQ, RT-qPCR analysis was performed using the SYBR Green Select method (SYBRTM Select Master Mix, Applied Biosystems, Waltham, MA, USA) and the comparative Ct method [29]. Total RNA was extracted, as described by Mani et al [9]. RNA was extracted from the frozen tissue (1 mg) stored at −80 °C using the Trizol method, described by the manufacturer (Invitrogen, Waltham, MA, USA). Treat samples with the DNase I kit (OMEGA, Norcross, GA, USA), as required by the manufacturer to avoid genomic contamination. The extracted RNA was quantified spectrophotometrically using Nanodrop 2000 (Thermo, Waltham, MA, USA). Samples with a 260/280 nm ratio below 1.8 would be re-extracted. The integrity of the extracted RNA was verified by electrophoresis. After extracting RNA from the small intestine of the same experimental animal, six genes were randomly selected for mRNA expression confirmation. The genes selected for RT-qPCR analysis were *CRYAB*, *PGM5, GCA*, *KRIT1*, *SLA-1,* and *HSPE1*. All qPCR primers used in this study are listed in Supplementary Table S2, in which the *ACTB* gene was used as an endogenous control. The sample RNA was reverse transcribed into cDNA, according to the instructions of the PrimeScript RT Reagent Kit (TAKARA, Kusatsu, Shiga, Japan). Subsequently, RT-qPCR was performed using a Qiagen Quantitative Reaction Kit (QuantiFast SYBR Green PCR Kit, Qiagen, Hilden, Germany), with three replicates each. The qPCR thermal cycling protocol was as follows: denaturation at 95 °C, hot start for 5 min; 45–50 PCR cycles (95 °C, 10 s, 60 °C, 15 s, 72 °C, 20 s); dissolution profile (95 °C, 15 s, 55 °C, 15 s, 95 °C, 15 s).

## 3. Results

### 3.1. Statistics and Analysis of FE-Related Phenotypes

**Figure 1:**
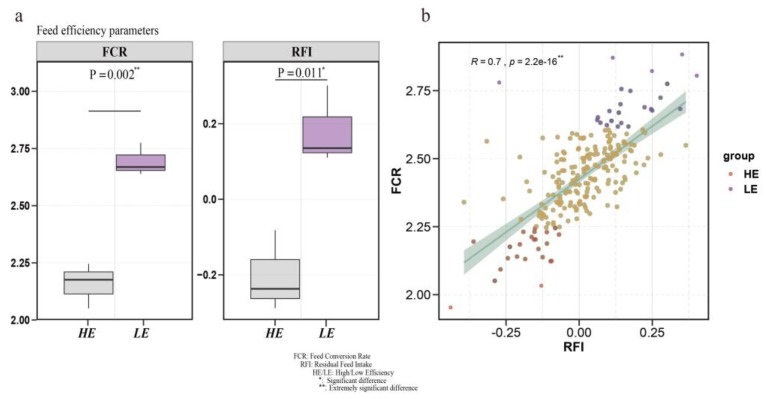
Statistics results and analysis of feed efficiency (FE)-related phenotypes. (**a**) The feed conversion ratio (FCR) and residual feed intake (RFI) between high- and low-FE groups. (**b**) Correlation analysis of RFI and FCR related to feed efficiency in 225 pigs.

FCR and RFI phenotypic data are often used to assess feed efficiency. We compared the FCR phenotypic data between high- and low-FE groups; the two groups had significant differences (*p* = 0.002) (Figure 1a). Then, we also compared RFI traits and found the same results (Figure 1a) (*p* = 0.011). Furthermore, to verify the correlation of RFI and FCR phenotypes, we performed a correlation analysis and found a moderately positive correlation between these two phenotypes (phenotypic correlation R = 0.7, *p* = 2.20 × 10^–16^) (Figure 1b). 

### 3.2. Protein Identification 

**Figure 2:**
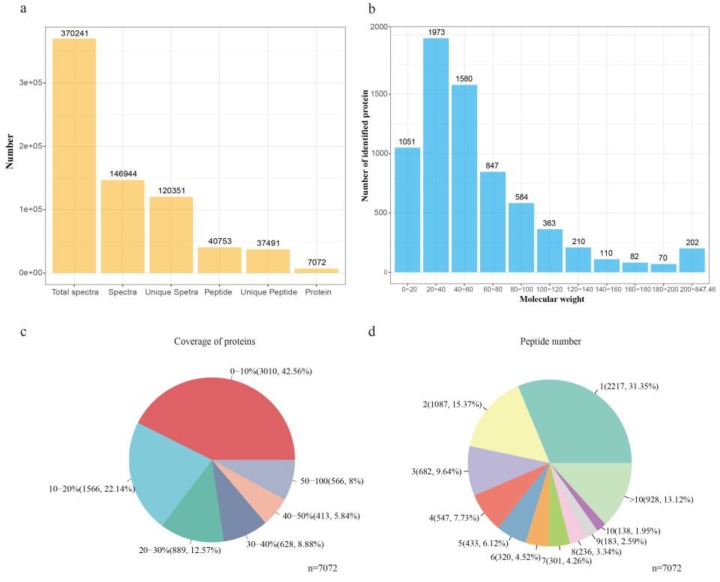
Protein identification and analysis using the isobaric tag for relative and absolute quantification (iTRAQ). (**a**) Overview of protein identification results. (**b**) Classification of identified proteins by molecular weight. (**c**) Coverage of proteins by the identified peptides. (**d**) Distribution of proteins with different peptide numbers.

A total of 370,241 spectra were generated, in which a total of 40,753 peptides and 7072 proteins were identified under the false discovery rate (FDR) ≤0.01 filtration standard (Figure 2a and Supplementary Table S3). Almost three-quarters of the identified proteins (77.08%) had molecular weights in the range of 0–20 kD (1051), 20–40 kD (1973), 40–60 kD (1580), and 60–80 kD (847) (Figure 2b). Moreover, 57% and 35% of the identified proteins showed more than 10% and 20% sequence coverage, respectively, indicating the high peptide coverage of the identified proteins (Figure 2c). Further, approximately 53.28% of the identified proteins had three or more peptides (Figure 2d).

### 3.3. Protein Quantification by iTRAQ Coupled with LC-MS/MS 

**Figure 3:**
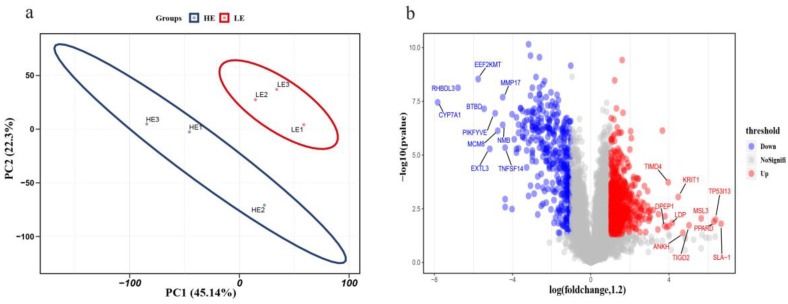
Differentially expressed protein analysis between high- and low-FE groups. (**a**) Principal component analysis of relative expression levels of proteins. The red and blue circles represent the low-FE and high-FE groups, respectively. (**b**) Volcano plot of differentially expressed proteins (DEPs) of the small intestine. The red and green dots represent up- and downregulated DEPs, respectively. Gray dots represent other detected proteins that do not meet the screening criteria.

We used the relative expression levels of each protein to perform principal component analysis (PCA) on the high-FE and low-FE data and found that the protein expression could be clearly distinguished into two groups (Figure 3a). The results proved that there were substantial differences between the high- and low-FE groups. The identified proteins were screened by fold-change greater than 1.2 or less than 0.84 with an unadjusted *p*-value less than 0.05 [25]. We obtained 1219 proteins that were significantly differentially expressed between the high- and low-FE pigs. Among 1219 differentially expressed proteins (DEPs), 785 were upregulated, and 484 downregulated in the high-FE group compared with the low-FE group (Supplementary Table S4). Upregulated, insignificant, and downregulated proteins of all identified proteins between the high- and low-FE groups were plotted in a volcano plot (Figure 3b). Correspondingly, the gene names of the top ten up- or downregulated proteins are shown separately in Figure 3b.

### 3.4. GO Annotation of DEPs

**Figure 4:**
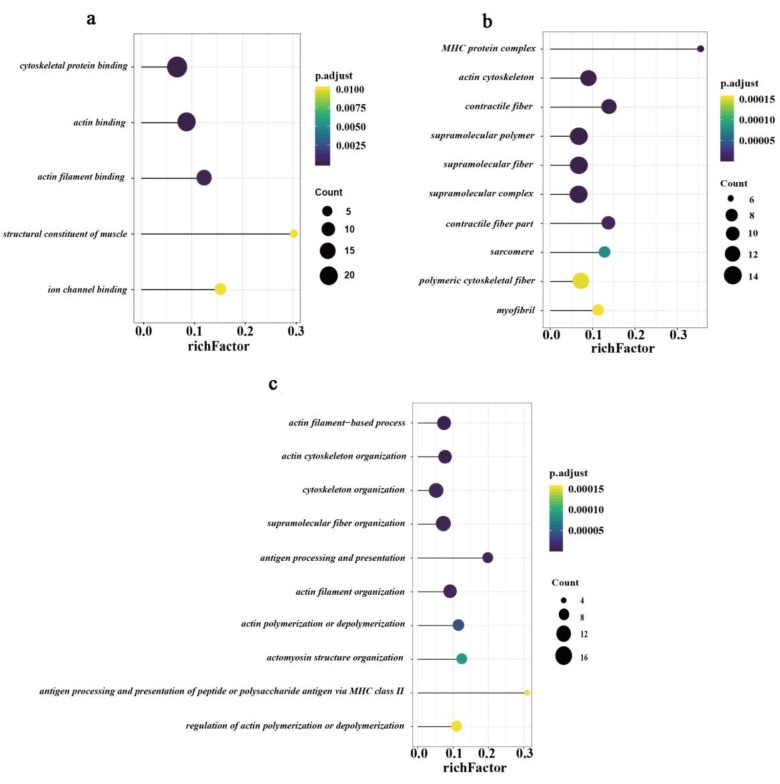
GO (gene ontology) enrichment annotation of all differentially expressed proteins. (**a**) All five GO terms for molecular function. (**b**) Top ten GO terms for the cellular component. (**c**) Top ten GO terms for biological process. The X-axis indicates a rich factor, and the Y-axis indicates terms. Colors of dots represent *p*-values, and sizes of dots represent the number of genes.

We performed gene ontology (GO) annotation analysis of DEPs using the ClusterProfiler R package [27]. With an adjusted *p*-value less than 0.05 as the threshold, a total of 73 GO terms were significantly enriched, including 38 biological process terms, 30 cellular component terms, and five molecular function terms (Supplementary Table S5). In the cellular component analysis, DEPs in the top ten terms were mainly located in myosin heavy chain (MHC) protein complex, actin cytoskeleton, contractile fiber, and supramolecular polymer (Figure 4a). Meanwhile, the top five GO terms for molecular function showed that the differentially expressed proteins were prominently involved in cytoskeletal protein binding, actin-binding, actin filament binding, structural constituent of muscle, and ion channel binding (Figure 4b). Similarly, the top ten terms of the biological process showed that most DEPs were enriched in actin filament-based process, actin cytoskeleton organization, cytoskeleton organization, actin filament organization, actin polymerization or depolymerization, and regulation of actin polymerization or depolymerization terms (Figure 4c).

### 3.5. KEGG and Reactome Pathway Analysis of the DEPs

**Figure 5:**
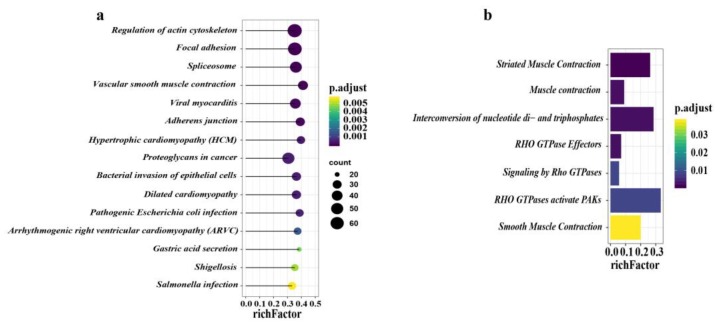
KEGG (Kyoto Encyclopedia of Genes and Genomes) and Reactome pathway annotation analysis. (**a**) The top 15 pathways of all DEPs in KEGG enrichment analysis. (**b**) All seven pathways of DEPs in Reactome pathway analysis. The X-axis indicates the rich factor, and the Y-axis indicates the pathway. Bar colors represent *p*-values.

In total, there were 1219 differentially expressed proteins located in KEGG, and 42 pathways were significantly enriched (adjusted *p* < 0.05) (Supplementary Table S6). The top 15 pathways of DEPs in the KEGG analysis are presented (Figure 5a). Most DEPs were mainly enriched in two major categories, namely, small intestine structure and small intestine movement. Pathways associated with small intestinal structures included regulation of actin cytoskeleton, focal adhesion, adherens junction, and tight junction pathways (Supplementary Table S6). Furthermore, the vascular smooth muscle contraction pathway was related to the movement of the small intestine.

In addition, based on the Reactome pathway analysis, a total of seven pathways were significantly enriched (Figure 5b). The majority of DEPs participated in striated muscle contraction, muscle contraction, interconversion of nucleotide di- and triphosphates, Rho GTPase effectors, signaling by Rho GTPases, Rho GTPase activation of p21-activated kinase (PAKs), and smooth muscle contraction (Supplementary Table S7). 

Based on GO functional analysis and KEGG pathway analysis, 40 DEPs in six pathways related to the small intestinal structure were selected to form an interaction network (Supplementary Figure S1). These six pathways included regulation of actin cytoskeleton, actin filament-based process, actin cytoskeleton organization, cytoskeleton organization, actin filament organization, and muscle structure development pathway. To visualize the relationship between these proteins and their functions, we then classified them by function and represented them in different colors of the node border. There were 18 DEPs involved in actin polymerization regulation, five related to cytoskeleton, three related to muscle contraction, and five involved in cell growth and development. 

### 3.6. Real-Time Quantitative Polymerase Chain Reaction

**Figure 6:**
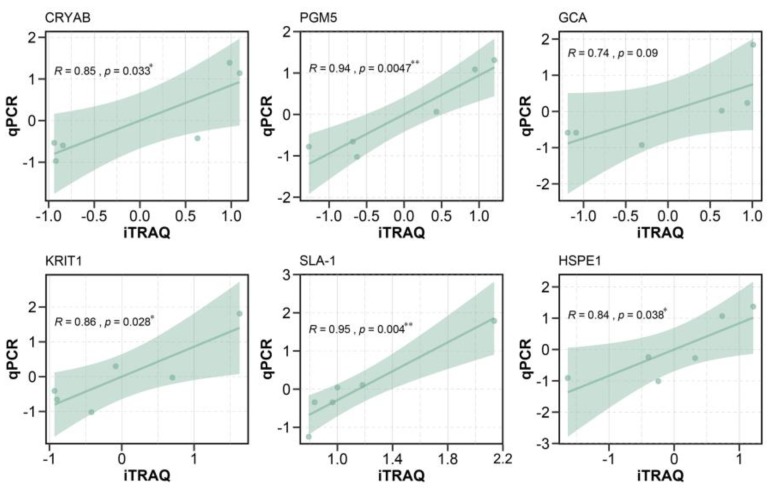
Correlation analysis between RT-qPCR results and iTRAQ. Results of correlation analysis between *CRYAB*, *PGM5*, *GCA*, *KRIT1*, *SLA-1,* and *HSPE1* gene expression and iTRAQ. The X-axis indicates a normalized fold change of protein in sequencing. The Y-axis indicates a normalized fold change of RT-qPCR.

To verify the DEPs identified by iTRAQ, we extracted RNA from the small intestine of the same experimental animals. Then, six genes corresponding to six DEPs were randomly selected for RT-qPCR, including *CRYAB*, *PGM5, GCA*, *KRIT1*, *SLA-1*, and *HSPE1*. Correlation analysis was performed between the expression levels of the six DEPs and the mean ratios obtained by iTRAQ (Figure 6). The results of RT-qPCR were exactly the same as those of iTRAQ.

## 4. Discussion

Although many omics methods have been used to study feed efficiency traits, very limited amounts of research have used the iTRAQ method to study this trait in DLY pigs at the protein level. In the present study, we compared the differentially expressed proteins between the high-FE and low-FE groups using iTRAQ. Our results provided a new perspective for exploring candidate genes that affect FE traits in DLY commercial pigs. To evaluate feed efficiency, previous studies have used two phenotypic traits, RFI and FCR, which have been shown to have high positive phenotype and genetic correlations (R = 0.67–0.89) [3,30,31,32]. In this study, we used the same calculation method to evaluate FE and found that the correlation between FCR and RFI was consistent with previous studies (R = 0.7). 

The digestion and absorption capacities of the small intestine are closely related to feed efficiency traits. Previous studies have shown that the small intestine structures of pigs, such as microvilli, focal adhesions, and intestinal mucosa, were important factors affecting the absorption of nutrients in the small intestine [33,34]. Previous research showed that epithelial paracellular permeability was regulated by the apical complex, whose main components are tight junctions and adherens junctions [35]. In another study, it was revealed that tight junctions acted as a selective permeability barrier and controlled the permeability of the intestinal mucosa, thereby selectively regulating the entry of small molecules and ions into the body [33,36]. Similarly, our study also found that many DEPs were significantly enriched in the adherens junction and tight junction pathway. Moreover, in the small intestine, G-actin forms a cylindrical fiber in the form of a spiral, actin filament, or microfilament, which is the basic structure for forming microvilli [37,38]. Correspondingly, our results found that many DEPs were involved in actin filament-based process, actin filament organization, actin polymerization or depolymerization, actin filament polymerization, and actin filament binding pathways. In these pathways, we found that hematopoietic lineage cell-specific (HCLS1), Ras homologous gene family, member A (RHOA) associated with actin filament formation, and ezrin (EZR) associated with actin cytoskeletal regulation were significantly upregulated in the high feed efficiency group. On the one hand, Uruno et al. [39] indicated that HCLS1 protein, which is encoded by the *HCLS1* gene, promoted the formation of branched actin filaments induced by the Arp2/3 complex. Another important protein that might affect the FE trait is RHOA, a small GTPase protein that is encoded by the *RHOA* gene and plays an important role in microvilli production [40]. The Rho family phosphorylates actin at the Thr-558 downstream of Rho, thereby structurally converting actin monomers to filaments [41,42]. Furthermore, studies have revealed that ezrin can directly participate in the morphogenesis of the free surface structure of the plasma membrane, especially the formation of intestinal epithelial microvilli, and can maintain the villus structure [43,44]. Therefore, *HCLS1*, *RHOA,* and *EZR* might be important candidate genes for affecting feed efficiency. On the other hand, by KEGG enrichment analysis, our results showed that in addition to RHOA, HCLS1, and EZR, cell division control protein 42 homolog (CDC42) and Ras-related C3 botulinum toxin substrate 1 (RAC1) were also upregulated in the high-FE group. Previous works have shown that CDC42 and RAC1 proteins, similar to RHOA and HCLS1, not only are regulators of the agonist cytoskeleton but also induce actin polymerization [45,46]. Moreover, our protein interaction network also indicated close associations between CDC42, RAC1, EZR, RHOA, and HCLS1 proteins, all of which are active participants in microvilli formation.

At present, no studies have considered *HCLS1*, *RHOA*, *CDC42*, *RAC1,* and *EZR* as potential genes affecting the feed efficiency of DLY pigs. Thus, we expect our findings to contribute to further understanding of the relationship between these five important proteins and feed efficiency in pigs. In addition, it is worth noting that our results could only provide preliminary insights into the study of feed efficiency, and our future research on the relationship between the small intestine and feed efficiency needs to be combined with multiple omics for analysis.

## Supplementary Materials

The following are available online at https://www.mdpi.com/2076-2615/10/2/189/s1, Figure S1: Interaction network of important proteins involved in central terms and pathways, Table S1: Metadata of experimental animals, Table S2: Primers for qPCR, Table S3: All identified proteins, Table S4: All DEPs, Table S5: Gene ontology enrichment result, Table S6: KEGG enrichment result and Table S7: ReactomePA pathway enrichment result.
