# Supplementary material for: Identification of Important Proteins and Pathways Affecting Feed Efficiency in DLY Pigs by iTRAQ-Based Proteomic Analysis
Source: Animals (Basel). 2020 Jan 22;10(2):189. doi: 10.3390/ani10020189 (PMC7070517; doi:10.3390/ani10020189)
Supplement: Supplementary file 1 [file animals-10-00189-s001.zip › Supplemental Figure S1, Table S1,S2,S5,S7.docx]

**Supplementary files: Identification of Important Proteins and Pathways Affecting Feed Efficiency in DLY Pigs by iTRAQ-Based Proteomic Analysis**

**Jie Wu ^1,$^, Xingwang Wang ^1,$^, Rongrong Ding ^1^, Jianping Quan ^1^, Yong Ye ^1^, Ting Gu ^1^, Zheng Xu ^1^, Enqin Zheng ^1^, Gengyuan Cai ^1^, Zhenfang Wu ^1^, Ming Yang ^2,^* and Jie Yang ^1,^***


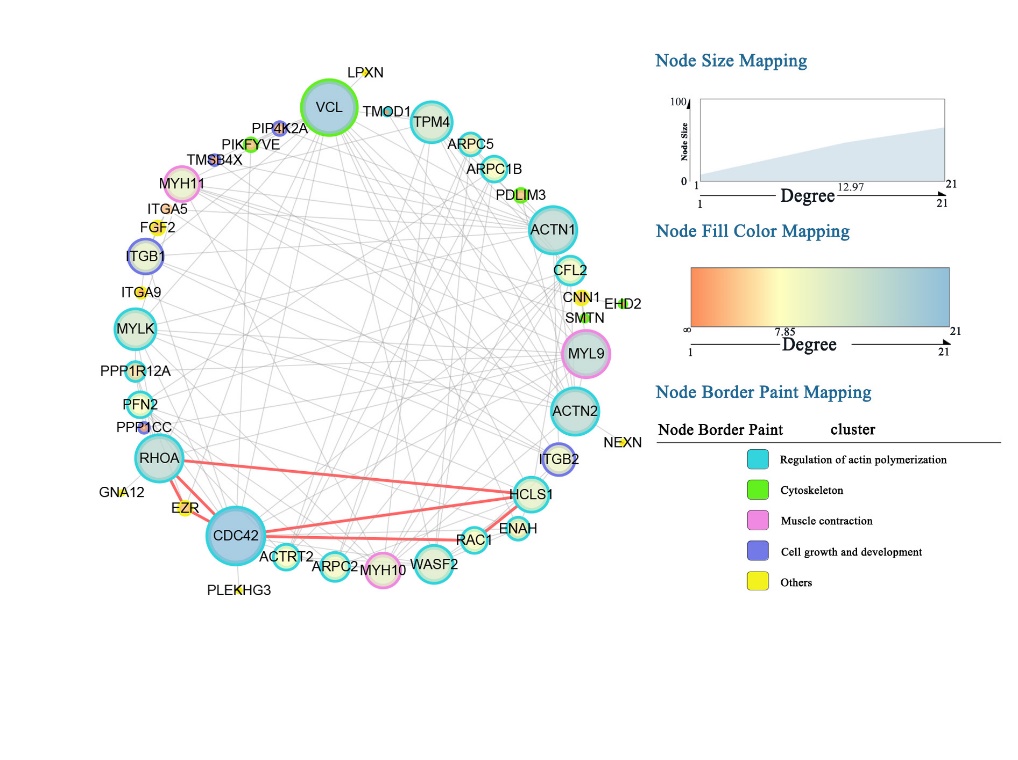


**Figure S1. Interaction network of important proteins involved in central terms and pathways.** Node size and node fill color indicate the degree of each DEP. Node border color indicates different functional classifications. Red lines indicate the interactions between five important proteins.

**Table S1.** Metadata of experimental animals.

| **Number** | **Sex** | **Starting weight/kg** | **Slaughter weight/kg** | **Starting age/day** | **Slaughter age/day** | **Feed efficiency** |
| --- | --- | --- | --- | --- | --- | --- |
| 1 | sow | 32.6 | 108.9 | 27 | 112 | High |
| 2 | sow | 33.1 | 118.4 | 27 | 99 | High |
| 3 | sow | 29.9 | 110.9 | 30 | 114 | High |
| 4 | sow | 29.8 | 107.6 | 32 | 117 | Low |
| 5 | sow | 29.8 | 97.7 | 37 | 126 | Low |
| 6 | sow | 29.4 | 98.2 | 34 | 125 | Low |

**Table S2.** Primers for qPCR.

| **Gene** | **F** | **R** | **tm(℃)** |
| --- | --- | --- | --- |
| GCA | CACTTGCACCTCCGCTTCTCTG | TTTCCGAACCCTCCTCCGTATCC | 60 |
| KRIT1 | TGGCAGAGGAGTATGAGCAGTGTC | CTTCACAGGCACTTCGATGGAGAG | 60 |
| PGM5 | ACGGAGGAAGACCACTCACCAAG | AGGCACAAACAGGTAGCAAGAAGG | 60 |
| HSPE1 | AGAAGTGCAGCTGAAACGGT | ACAGCTACTACGGTTGCTTGC | 60 |
| CRYAB | AGAGATGCGTCTGGAGAAGGACAG | CCCAACACCTTGACCTTGAGTTCC | 60 |
| SLA-1 | GCCAGGACCAGAGCCAGGAC | CTCTCCAGGAGGCACCACCAG | 60 |
| ACTB | CCACGAGACCACCTTCAACTC | TGATCTCCTTCTGCATCCTGT | 60 |

**Table S5.** Gene ontology enrichment result.

| **ID** | **Description** | **GeneRatio** | **BgRatio** | ***p*-value** | ***p*-adjust** | ***q*-value** | **geneID** | **Count** | **richFactor** |
| --- | --- | --- | --- | --- | --- | --- | --- | --- | --- |
| GO:0008092 | cytoskeletal protein binding | 20/77 | 282/6500 | 4.51E-11 | 1.02E-08 | 9.45E-09 | CNN1/RHOA/TAGLN/TPM4/PDLIM3/DSTN/CFL2/TMSB4X/FYN/TPPP/ACTN2/KIFAP3/TMOD1/GJA1/FLNC/KIF2B/HCLS1/BAG2/PFN2/HSPB7 | 20 | 0.070922 |
| GO:0003779 | actin binding | 11/77 | 129/6500 | 2.69E-07 | 3.03E-05 | 2.82E-05 | CNN1/TAGLN/TPM4/DSTN/CFL2/TMSB4X/ACTN2/TMOD1/FLNC/HCLS1/PFN2 | 11 | 0.085271 |
| GO:0051015 | actin filament binding | 7/77 | 58/6500 | 4.60E-06 | 0.000345 | 0.000321 | TAGLN/TPM4/DSTN/CFL2/ACTN2/TMOD1/FLNC | 7 | 0.12069 |
| GO:0008307 | structural constituent of muscle | 3/77 | 10/6500 | 0.000181 | 0.01005 | 0.009356 | MYL6/TPM4/PDLIM3 | 3 | 0.3 |
| GO:0044325 | ion channel binding | 4/77 | 26/6500 | 0.000223 | 0.01005 | 0.009356 | FYN/KCNIP2/ACTN2/BAG2 | 4 | 0.153846 |

**Table S7.** ReactomePA pathway enrichment result.

| **ID** | **Description** | **GeneRatio** | **BgRatio** | ***p*-value** | ***p*-adjust** | ***q*-value** | **geneID** | **Count** | **richFactor** |
| --- | --- | --- | --- | --- | --- | --- | --- | --- | --- |
| R-SSC-390522 | Striated Muscle Contraction | 5/55 | 19/4219 | 3.17E-06 | 0.000982 | 0.000904 | 396725/397608/100154254/100157406/100316850 | 5 | 0.263158 |
| R-SSC-397014 | Muscle contraction | 8/55 | 88/4219 | 1.42E-05 | 0.002203 | 0.002027 | 396725/396807/397608/100037948/100154254/100157406/100157760/100316850 | 8 | 0.090909 |
| R-SSC-499943 | Interconversion of nucleotide di- and triphosphates | 4/55 | 14/4219 | 2.35E-05 | 0.002334 | 0.002148 | 397463/100157205/100521423/100621123 | 4 | 0.285714 |
| R-SSC-195258 | RHO GTPase Effectors | 9/55 | 127/4219 | 3.01E-05 | 0.002334 | 0.002148 | 396807/396849/396970/397610/733585/733612/100157760/100520975/100621523 | 9 | 0.070866 |
| R-SSC-194315 | Signaling by Rho GTPases | 9/55 | 157/4219 | 0.000159 | 0.00861 | 0.007923 | 396807/396849/396970/397610/733585/733612/100157760/100520975/100621523 | 9 | 0.057325 |
| R-SSC-5627123 | RHO GTPases activate PAKs | 3/55 | 9/4219 | 0.000167 | 0.00861 | 0.007923 | 396807/733585/100157760 | 3 | 0.333333 |
| R-SSC-445355 | Smooth Muscle Contraction | 3/55 | 15/4219 | 0.000854 | 0.037816 | 0.034798 | 396807/397608/100157760 | 3 | 0.2 |
